# Web GIS in practice IV: publishing your health maps and connecting to remote WMS sources using the Open Source UMN MapServer and DM Solutions MapLab

**DOI:** 10.1186/1476-072X-5-6

**Published:** 2006-01-18

**Authors:** Maged N Kamel Boulos, Kiyoshi Honda

**Affiliations:** 1 Faculty of Health and Social Work, University of Plymouth, Drake Circus, Plymouth, Devon PL4 8AA, UK; 2 Remote Sensing and GIS Field of Study, Asian Institute of Technology (AIT), Pathumthani 12120, Thailand

## Abstract

Open Source Web GIS software systems have reached a stage of maturity, sophistication, robustness and stability, and usability and user friendliness rivalling that of commercial, proprietary GIS and Web GIS server products. The Open Source Web GIS community is also actively embracing OGC (Open Geospatial Consortium) standards, including WMS (Web Map Service). WMS enables the creation of Web maps that have layers coming from multiple different remote servers/sources. In this article we present one easy to implement Web GIS server solution that is based on the Open Source University of Minnesota (UMN) MapServer. By following the accompanying step-by-step tutorial instructions, interested readers running mainstream Microsoft^® ^Windows machines and with no prior technical experience in Web GIS or Internet map servers will be able to publish their own health maps on the Web and add to those maps additional layers retrieved from remote WMS servers. The 'digital Asia' and 2004 Indian Ocean tsunami experiences in using free Open Source Web GIS software are also briefly described.

## Background

One of the most important powers of GIS is the capability to publish and share geo-spatial information on the Internet among large numbers of people. Sharing of geo-spatial information is an important and effective way of working in many kinds of applications. Geo-spatial information includes not only maps or locations of landmarks/facilities, but multiple attribute data, socio-economic data, ground photos, aerial photographs, satellite images, etc., which may have static or dynamic characteristics. By sharing this information on the Internet, accessibility, time response, and understandability are drastically improved compared to conventional paper distribution of maps or character based Web systems. Users will have more freedom to choose information or layers to see and synthesize maps that will fit their own requirements.

### The growing interest in Open Source GIS and Web GIS

Interest in Open Source GIS and Web GIS software has grown considerably over the past few years (see definition of Open Source at [1]). Dedicated Web portals like [2-4] offer users many Open Source GIS and Web GIS software options to choose from. These include Quantum GIS [5], a user friendly Open Source desktop GIS that runs on Linux, Unix, Mac OSX, and Windows, and the now very popular University of Minnesota (UMN) MapServer [6] and related applications, which are the focus of this article.

The Open Source GIS/Web GIS community is very actively embracing the Open Geospatial Consortium (OGC [7]) standards; for example, UMN MapServer already supports several OGC Web specifications, including WMS (Web Map Service – client/server), non-transactional WFS (Web Feature Server – client/server) and GML (Geography Markup Language).

Today one can say without exaggeration that Open Source GIS and Web GIS software have reached a stage of maturity, sophistication, robustness and stability, and usability and user friendliness that parallels, if not (sometimes) exceeds, that of commercial, proprietary GIS and Web GIS products. Commercial software manufacturers are even starting to back Open Source. Recently, the MapServer Community and Autodesk announced the new 'MapServer Foundation' [8-10].

A related international conference, *Geoinformatics 2006 – Free and Open Source software: 12–15 September 2006; Lausanne, Switzerland *[11], has been announced in 2005. The event is concerned with addressing geospatial data technologies developed by, or of relevance to, the Open Source community.

### On UMN MapServer, DM Solutions MapLab, and WMS sources

In this article we present one easy-to-implement Web GIS solution that is based on UMN MapServer. By following the accompanying step-by-step tutorial instructions, interested readers using mainstream Microsoft^® ^Windows machines and with no prior experience in Web GIS or Internet map servers will be able to publish their own health maps on the Web and add to those maps additional layers retrieved from remote WMS servers (see additional file 1: Step-by-step tutorial – publishing your maps and connecting to remote WMS sources using the Open Source UMN MapServer and DM Solutions MapLab).

MapServer is an Open Source development environment for constructing spatially enabled Internet-Web applications. Readers can browse the 'MapServer application gallery' [12] for some interesting examples. There is also a Tsunami Web Map Server developed by Professor Kiyoshi Honda and colleagues using the Open Source UMN MapServer [13-15] (see also 'Results and discussion' section below for further information about the Tsunami Web Map Server).

MapServer, through the use of special libraries, can access various raster and vector data formats without data conversion [16]. MapServer features and documentation can be found at [6].

MapLab from DM Solutions Group Inc., Canada, is an Open Source suite of effective and intuitive Web-based tools to create and manage UMN MapServer Web mapping applications and map files. It consists of three components: MapEdit, MapBrowser and GmapFactory [17,18].

**Figure 1:**
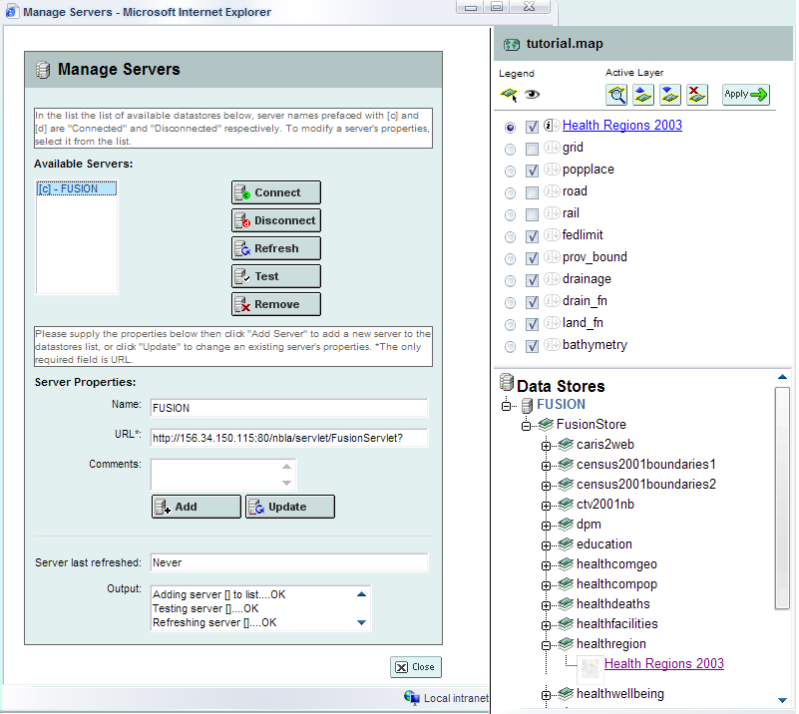
**Connecting to a remote WMS source in MapBrowser**. Left: screenshot of the 'Manage Servers' box in MapBrowser, which is used to add/connect to remote WMS servers. In this screenshot, 'FUSION', the 'Environmental Health Mapping Portal WMS' (), which is hosted by the New Brunswick Lung Association, has been added. Right: screenshot of part of the main MapBrowser page showing the layers retrieved from the remote WMS server we have connected to in the 'Manage Servers' box. One of these layers, 'Health Regions 2003', has been selected and added to our local 'tutorial.map'.

MapEdit is a visual administration tool for the editing, validation and management of map files. MapBrowser is a complementary tool for the visual selection of spatial data from local and remote WMS sources (Figure 1), while GMapFactory is the final-step tool used for the rapid creation and deployment of Web mapping applications, e.g., to define the layout of an application and specify which mapping interface components to include.

OGC WMS provides operations in support of the creation and display of registered and superimposed map-like views of information that come simultaneously from multiple servers/sources that are both remote and heterogeneous. WMS thus enables a distributed network of interoperable geospatial information providers and the creation of Web maps that have layers coming from multiple different remote servers/sources [13] (see also WMS implementation specification at [19]).

With the introduction of WMS specification and also OGC Web Feature Service (WFS – see [20]), it has become easy to publish and share any geo-spatial information on the Internet. WMS, which is currently popular in actual applications, basically creates maps (PNG – Portable Network Graphics, or JPEG formats) of the requested area, which standard browsers can render. Thus, users do not have to copy huge data sets to local systems. WFS supplies users with only the geographic features that satisfy their filtering criteria.

WMS server lists and discovery portals are available on the Web to help users locate and connect (using software tools implementing the OGC WMS specification) to suitable data sources for their maps, e.g., [21,22] to name two such services.

In a previous article in this 'Web GIS in practice' series [23], we explored mapping solutions from Google and MSN. It is noteworthy that Google Earth also currently supports the import of WMS data into its enterprise client [24]. Users can subscribe to a WMS server and see that as an overlay on the Google Earth data as they pan around [25].

## Methods

Technical expertise is generally required to install, customize and manage an Internet map server, whether it is a commercial product or an Open Source one. However, in this article we introduce an easy-to-follow, illustrated step-by-step tutorial for installing and using such servers on machines running Microsoft^® ^Windows (see additional file 1: Step-by-step tutorial – publishing your maps and connecting to remote WMS sources using the Open Source UMN MapServer and DM Solutions MapLab).

The tutorial in the accompanying 'additional file 1' is based on the MS4W package (MapServer for Microsoft^® ^Windows [26]) and a matching MapLab installation packaged for MS4W (the latest versions of both packages and other additional packages can be downloaded at [27,28]).

MS4W has been prepared by Jeff McKenna of DM Solutions Group Inc., Canada. The basic MS4W package installs a pre-configured Web Server environment that includes the following components:

• Apache HTTP Server [29];

• PHP [30];

• MapServer CGI (Common Gateway Interface);

• PHP/MapScript [31,32];

• GDAL (Geospatial Data Abstraction Library)/OGR Utilities [33-36]: GDAL is a translator library for raster geospatial data formats that is released under an Open Source license. As a library, it presents a single abstract data model to the calling application for all supported formats. The related OGR library (which lives within the GDAL source tree) provides a similar capability for simple features vector data [16]. UMN MapServer can access TIFF/GeoTIFF, EPPL7 [37], and many other formats via GDAL, and ESRI Shapefiles, PostGIS, ESRI ArcSDE, Oracle Spatial, MySQL and many others via OGR;

• MapServer Utilities;

• OGR/PHP Extension; and

• OWTChart: The OWTChart Engine produces GIF images of virtually any type of chart from a set of input parameters. The program can be used as a CGI in a Web server environment [38].

## Results and discussion

**Figure 2:**
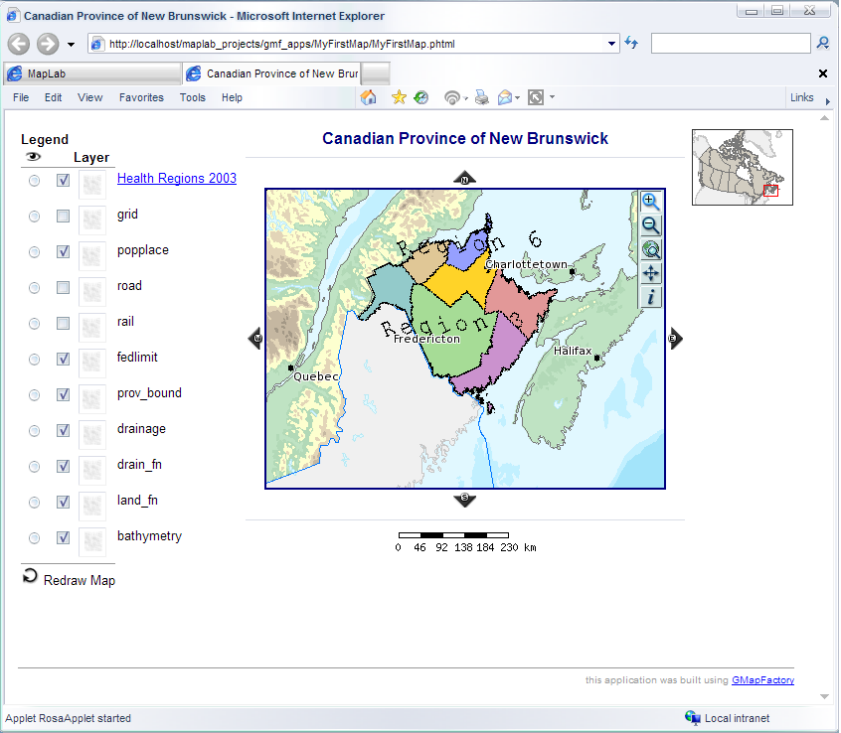
**Web map screenshot of the 'Canadian Province of New Brunswick' published using MapServer and MapLab**. Screenshot of a sample Web map of the 'Canadian Province of New Brunswick' that has been produced and published by following the instructions in the accompanying step-by-step tutorial (see additional file 1: Step-by-step tutorial – publishing your maps and connecting to remote WMS sources using the Open Source UMN MapServer and DM Solutions MapLab). The 'Health Regions 2003' layer in this map comes from a remote WMS source, while the other layers in the same map, e.g., 'popplace', are locally hosted on the same machine used to execute the tutorial.

Figure 2 shows a sample Web map of the 'Canadian Province of New Brunswick' that has been produced and published by following the instructions in the accompanying step-by-step tutorial (see additional file 1: Step-by-step tutorial – publishing your maps and connecting to remote WMS sources using the Open Source UMN MapServer and DM Solutions MapLab). The 'Health Regions 2003' layer in this map comes from a remote WMS source, the 'Environmental Health Mapping Portal WMS', which is hosted by the New Brunswick Lung Association (more information about this particular WMS server can be found at [39,40]). The other layers in the same map, e.g., 'popplace', ship with the MapLab package used in our tutorial, and are locally hosted on the same machine used to execute the tutorial.

### Other easy-to-use Open Source Web GIS installations for Windows based on UMN MapServer

These include CartoWeb [41], developed by Camptocamp SA, Switzerland, and the 'One Click Installation CD-ROM' kit of Professor Kiyoshi Honda of AIT, Thailand (see [42] and below).

### The 'digital Asia' and 2004 Indian Ocean tsunami experiences in FOSS

In 2002, Honda *et al*. [43] realized the importance of the sharing of geo-spatial information and proposed a mechanism called 'digital Asia' for implementation in Asia. A WMS for full resolution Landsat images has been developed under this concept [44].

Santitamnont *et al*. [45] reported the high potential of Free Open Source Software (FOSS) for sharing geo-spatial information in the context of the 'digital Asia' concept. There are lots of benefits of FOSS. Most probably the biggest benefit is the cost saving, but benefits are not limited to this only. FOSS software systems are being developed by many individual/company developers, thus bug fixing and update speeds are usually very fast. Any user can have access to the source code. This makes it possible for everyone to understand the algorithm and implementation, and, if they wish, also participate in the development of the software. The licensing scheme defined as GNU Public License (GPL) accelerates the expansion of FOSS community. There are several Open Source implementation of WMS and WFS such as the above mentioned UMN MapServer, and GeoServer [46].

In response to the Indian Ocean tsunami disaster on 26th December 2004, there was an urgent need to develop a framework for sharing not only maps but also remote sensing images, aerial photographs, field survey photos/movies, survey records, and socio-economic data. Tsunami relief works, such as damage mapping, infrastructure rehabilitation planning, hazard map development, etc., require these data. The benefits of the sharing include saving time for data collection, reducing duplicate efforts, providing better understanding of the damage, thus speeding up relief works. Honda *et al*. [13] developed a prototype within three weeks after the Indian Ocean tsunami disaster using FOSS. The data on the site were contributed by 14 organizations who shared their data sets (successful data sharing is very important in emergency responses and disaster informatics). One of the important features of WMS is that it is possible to link WMS servers to each other and share map layers between them. Data owners may keep their original data on their local servers, serving their data as images overlaid with layers from other WMS sources. All of this is very transparent to end users to the extent that some of them might think that all the map layers they are seeing are being served from the same single server they are accessing. This feature is a key enabler in the 'digital Asia' scheme and the above mentioned prototype for the tsunami disaster through its potentials for easing copyright issues and distributing the burden of database management.

However, the actual installation of FOSS-based WMS on to a server is not so simple, especially for beginners. While user manuals for individual packages are detailed enough, it is still difficult for users to manipulate a collection of FOSS, partly because of software dependencies. It was decided to develop a collection of FOSS for WMS installation as a kind of 'One Click Installation CD-ROM' for Microsoft^® ^Windows environment since the great majority of beginners wanting to experiment with FOSS Web GIS are using Windows [47]. Unified instructions for this FOSS collection were also developed and included on the same CD. They are based on DM Solutions kit [48] and instructions for Linux environment by Raghavan *et al*. [49]. Web GIS workshops were organized based on this CD-ROM at various conferences (AFITA in 2004 [50] and the First International Symposium on Health GIS in 2005 [51]), and attracted a lot of participants, who were interested in trying the WMS capability by themselves. The CD image is freely available from [52].

The use of mobile devices will further expand the efficiency and utility of WMS. Maps and data can now be retrieved using PDA devices in the field, and surveyors using such devices in the field are able to upload/update maps or other data on remote servers, which can then be shared immediately. Mobile clients present different processing challenges from desktop Web browsers. The location of a mobile device (taken from a GPS device) should be included in requests from the device to servers, so that users can immediately access the data that are specific to their current position. Ninsawat *et al*. [53] have demonstrated a mobile system to access their WMS server over a GPRS (General Packet Radio Service) mobile phone system in Thailand. To distinguish different users, they have built into their system a password authorization module using session variable and MD5 (Message-Digest Algorithm 5) encryption.

Publishing and sharing geo-spatial data in the health sector are increasingly becoming important and popular tasks in various applications. For example, health care organizations need to study how disease spreads, or how toxic substances affect human health, while health planners need to access details about how well patients are served by doctors at individual sites [54]. WMS and WFS can contribute to these information needs and applications by enabling the publication and sharing of geo-spatial information. The aforementioned Tsunami Web Map Server example demonstrates that the technical solutions provided by FOSS systems have very well matured and are now ready for use in actual mission critical applications in the health sector.

## Conclusion

Open Source Web GIS software systems have reached a stage of maturity, sophistication, robustness and stability, and usability and user friendliness rivalling that of commercial, proprietary GIS and Web GIS server products. The Open Source Web GIS community is also actively embracing OGC standards, including WMS. WMS enables the creation of Web maps that have layers coming from multiple different remote servers/sources.

In this article we presented one easy to implement Web GIS server solution that is based on the Open Source UMN MapServer. A step-by-step tutorial accompanies this article to help interested readers running mainstream Microsoft^® ^Windows machines, and with no prior technical experience in Web GIS or Internet map servers, to publish their own health maps on the Web and add to those maps additional layers retrieved from remote WMS servers.

The 'digital Asia' and 2004 Indian Ocean tsunami experiences in using free Open Source Web GIS software were also briefly discussed.

## Authors' contributions

MNKB conceived and drafted this manuscript and the accompanying step-by-step PDF tutorial. KH contributed important and unique insight to the article and wrote parts of it. Both authors read and approved the final manuscript.

## Supplementary Material

Additional File 1 **Step-by-step tutorial – publishing your maps and connecting to remote WMS sources using the Open Source UMN MapServer and DM Solutions MapLab**. An illustrated step-by-step tutorial to help interested readers running mainstream Microsoft^® ^Windows machines, and with no prior experience in Web GIS or Internet map servers, to publish their own health maps on the Web and add to those maps additional layers retrieved from remote WMS servers. Click here for file

## References

[B1] Definition of Open Source. http://www.opensource.org/docs/definition.php.

[B2] MapTools.org. http://www.maptools.org/.

[B3] Refractions Research. http://www.refractions.net/.

[B4] Open Source GIS. http://www.opensourcegis.org/.

[B5] Quantum GIS (QGIS). http://www.qgis.org/.

[B6] University of Minnesota (UMN) MapServer. http://mapserver.gis.umn.edu/.

[B7] Open Geospatial Consortium. http://www.opengeospatial.org/.

[B8] Schutzberg A MapServer Community, Autodesk Announce MapServer Foundation. Directions Magazine.

[B9] Autodesk Goes Open Source. http://www.autodesk.com/hpk-open_source.

[B10] MapServer Foundation. http://www.mapserverfoundation.org/.

[B11] Geoinformatics 2006 – Free and Open Source software (Conference). http://www.foss4g2006.org/.

[B12] UMN MapServer Gallery. http://mapserver.gis.umn.edu/gallery.html.

[B13] Honda K, Ninsawat S (2005). A WMS Server for Tsunami Geo-Spatial Information Sharing. Proceedings of First International Symposium on Area Informatics 2005 – Potential of GIS/RS in Area Studies: 24 March 2005; Asian Institute of Technology, IntERLab, Thailand.

[B14] (2005). Tsunami Web Mapserver (WMS). http://mapserver.hondalab.star.ait.ac.th/tsunami/.

[B15] Tsunami Disaster Mapping for Indian Ocean Coastal Regions. http://mapsherpa.com/tsunami/.

[B16] Mitchell T Web Mapping Illustrated (Using Open Source GIS Toolkits).

[B17] MapLab at MapTools.org. http://www.maptools.org/maplab/.

[B18] MapLab – MapEdit, MapBrowser, GMapFactory (DM Solutions site). http://www.dmsolutions.ca/technology/maplab.html.

[B19] WMS Implementation Specification. http://portal.opengeospatial.org/files/?artifact_id=5316.

[B20] WFS Implementation Specification. http://portal.opengeospatial.org/files/?artifact_id=8339.

[B21] Public OGC WMS Server List. http://www.skylab-mobilesystems.com/en/wms_serverlist.html.

[B22] GeoConnections Discovery Portal. http://geodiscover.cgdi.ca/gdp/search?action=executeSearch&entryType=service&portal=gdp&serviceType=Environment.

[B23] Boulos MN (2004). Web GIS in practice III: creating a simple interactive map of England's Strategic Health Authorities using Google Maps API, Google Earth KML, and MSN Virtual Earth Map Control. Int J Health Geogr.

[B24] Google Earth enterprise. http://earth.google.com/earth_enterprise.html.

[B25] Luccio M Google Earth Responds. GIS Monitor.

[B26] MapServer for Windows – MS4W. http://www.maptools.org/ms4w/.

[B27] MS4W Downloads (1). http://www.maptools.org/ms4w/index.phtml?page=downloads.html.

[B28] MS4W Downloads (2). http://dl.maptools.org/dl/ms4w/.

[B29] The Apache Software Foundation. http://www.apache.org/.

[B30] PHP: Hypertext Preprocessor. http://www.php.net/.

[B31] PHP MapScript at MapTools.org. http://www.maptools.org/php_mapscript/.

[B32] PHP/Mapscript Class Reference. http://mapserver.gis.umn.edu/doc42/phpmapscript-class-guide.html.

[B33] GDAL – Geospatial Data Abstraction Library. http://www.gdal.org/.

[B34] GDAL Raster Formats. http://gdal.maptools.org/formats_list.html.

[B35] OGR Simple Feature Library. http://www.gdal.org/ogr/.

[B36] OGR Vector Formats. http://ogr.maptools.org/ogr_formats.html.

[B37] EPPL7. http://www.lmic.state.mn.us/EPPL7/EPPL7/.

[B38] OWTChart. http://www.maptools.org/owtchart/index.phtml.

[B39] The New Brunswick Lung Association's Environmental Health Mapping Project. http://www.nb.lung.ca/mapping/.

[B40] Environmental Health Mapping Portal (GeoConnections Discovery Portal). http://geodecouverte.icdg.ca/gdp/search?action=entrySummary&entryType=service&entryId=5157&entryLang=en&portal=gdp.

[B41] CartoWeb. http://www.cartoweb.org/.

[B42] Publishing Your GIS Data on the Web Using FOSS. http://dl.maptools.org/dl/maplab/mapserver-foxserv-install.pdf.

[B43] Honda K, Raghavan V, Santitamnont P, Lertlum S Digital Asia: development of the data-sharing mechanism for geo-spatial information in Asia. Proceedings of the 23rd Asian conference on Remote Sensing: 25–29 November 2002; Kathmandu, Nepal.

[B44] Santitamnont P, Raghavan V, Honda K Open Source Software Solutions and their Potential for Spatial Data Infrastructure Development. Proceedings of the 23rd Asian conference on Remote Sensing: 25–29 November 2002; Kathmandu, Nepal.

[B45] Ninsawat S, Honda K, Horanont T, Yokoyama R, Inex AVM (2003). Remote Sensing Image Server based on WMS for GMS (Greater Mekong Sub-Region) Countries. Proceedings of the 24th Asian Conference on Remote Sensing: 3–7 November 2003, Busan, Korea CD-ROM; (FA5 Spatial Data Infrastructure 2).

[B46] (2003). GeoServer. http://docs.codehaus.org/display/GEOS/Home.

[B47] Honda K, Ninsawat S Publishing Your GIS Data on the Web Using Free and Open Source Software. Proceedings of AFITA2004, 4th Asian Conference for Information Technology in Agriculrue: 9–14 August 2004; Bangkok, Thailand.

[B48] (2004). DM Solutions Group – The Source for MapServer Solutions. http://www.dmsolutions.ca/.

[B49] Raghavan V, Santitamnont P, Masumoto S (2003). Training Notes on Spatial Data Sharing using Free and Open Source Software.

[B50] (2003). AFITA 2004 (Conference). http://www.jsai.or.jp/afita/#CONF.

[B51] (2003). First International Symposium on Health GIS 2005. http://www.j-geoinfo.net/HealthGIS/Main.htm.

[B52] (2003). Kiyoshi Honda homepage/contact information. http://www.rsgis.ait.ac.th/~honda.

[B53] Ninsawat S, Honda K (2004). The Application of GMS Remote Sensing Image Server for Mobile Devices. Proceedings of the 25th Asian Conference on Remote Sensing: 22–26 November 2004; Chiang Mai, Thailand.

[B54] Boulos MN, Roudsari AV, Carson ER (2001). Health Geomatics: An Enabling Suite of Technologies in Health and Healthcare (Methodological Review). J Biomed Inform.

