# Supplementary material for: Web GIS in practice IV: publishing your health maps and connecting to remote WMS sources using the Open Source UMN MapServer and DM Solutions MapLab
Source: Int J Health Geogr. 2006 Jan 18;5:6. doi: 10.1186/1476-072X-5-6 (PMC1352347; doi:10.1186/1476-072X-5-6)
Supplement: Additional File 1 — Step-by-step tutorial – publishing your maps and connecting to remote WMS sources using the Open Source UMN MapServer and DM Solutions MapLab. An illustrated step-by-step tutorial to help interested readers running mainstream Microsoft® Windows machines, and with no prior experience in Web GIS or Internet map servers, to publish their own health maps on the Web and add to those maps additional layers retrieved from remote WMS servers. [file 1476-072X-5-6-S1.pdf]

Step-by-step tutorial: publishing your maps and connecting to remote WMS sources using the Open Source UMN MapServer and DM Solutions MapLab

Prepared by: Maged N Kamel Boulos, PhD (mnkamelboulos@plymouth.ac.uk)

1. Download 'ms4w_1.3.0.zip' from http://dl.maptools.org/dl/ms4w/ms4w_1.3.0.zip and extract it to C:\ (it must be to the *root* of any of your hard drives; it doesn't have to be the C: drive); this will automatically create a folder 'ms4w' in C:\ and all subfolders and files in the zip archive will be extracted into 'ms4w'.

2. Download 'maplab_ms4w-2.2.1.zip' from http://dl.maptools.org/dl/ms4w/maplab_ms4w-2.2.1.zip and extract it also to C:\ (root of the drive where you have extracted the first archive in step 1 above); this will automatically use the folder 'ms4w' previously created in step 1 and all subfolders and files in the second zip archive will be extracted into 'ms4w'.

3. To start the Apache HTTP server, browse to 'C:\ms4w' (where all subfolders and files have been extracted in steps 1 and 2 above), and locate and run 'apache-install.bat' in that folder (just click or double-click 'apache-install.bat' to run it).

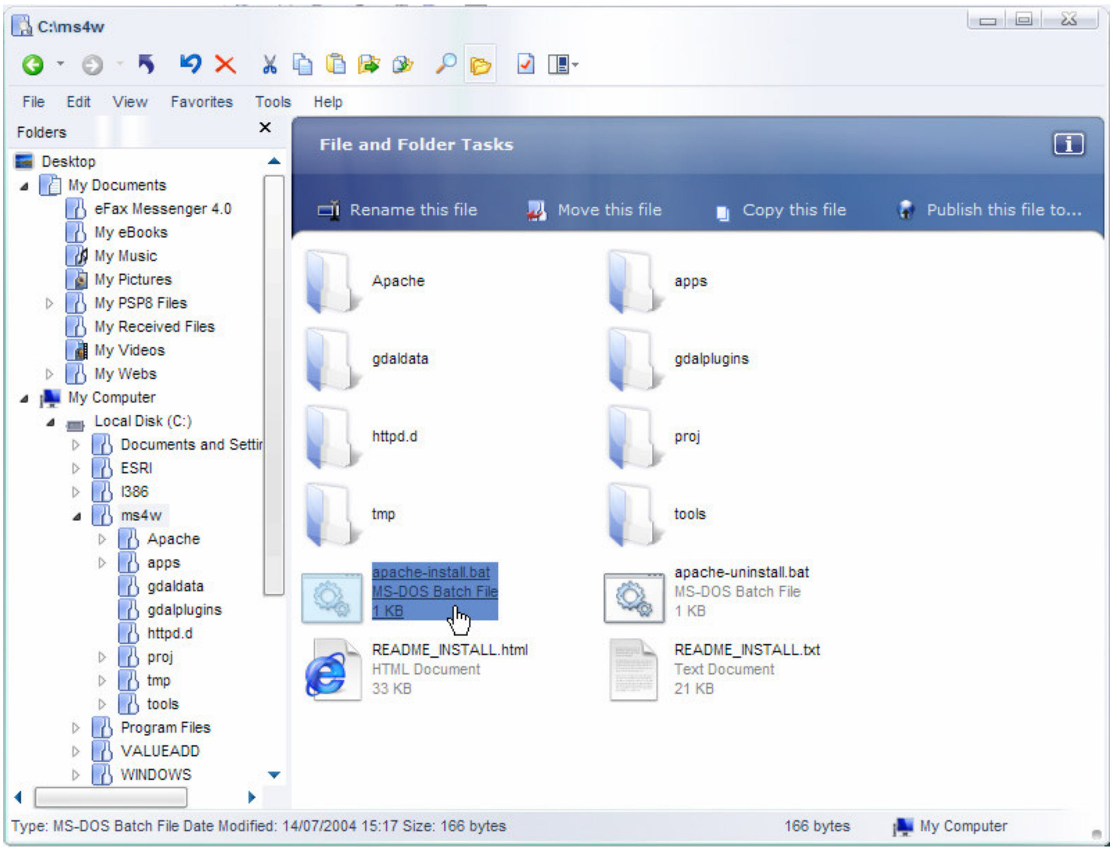

3. If you receive a 'Windows AntiSpyware Notice', choose 'Allow' and tick 'Remember this ction'. If you receive a 'Windows Security Alert' (Firewall) warning, choose 'Unblock'.

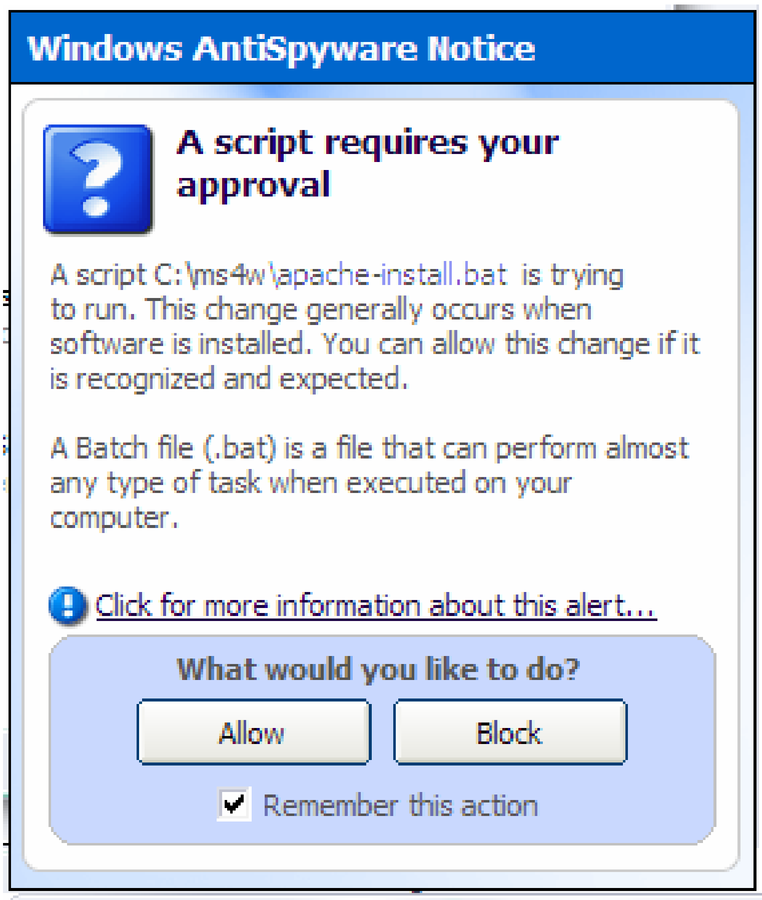

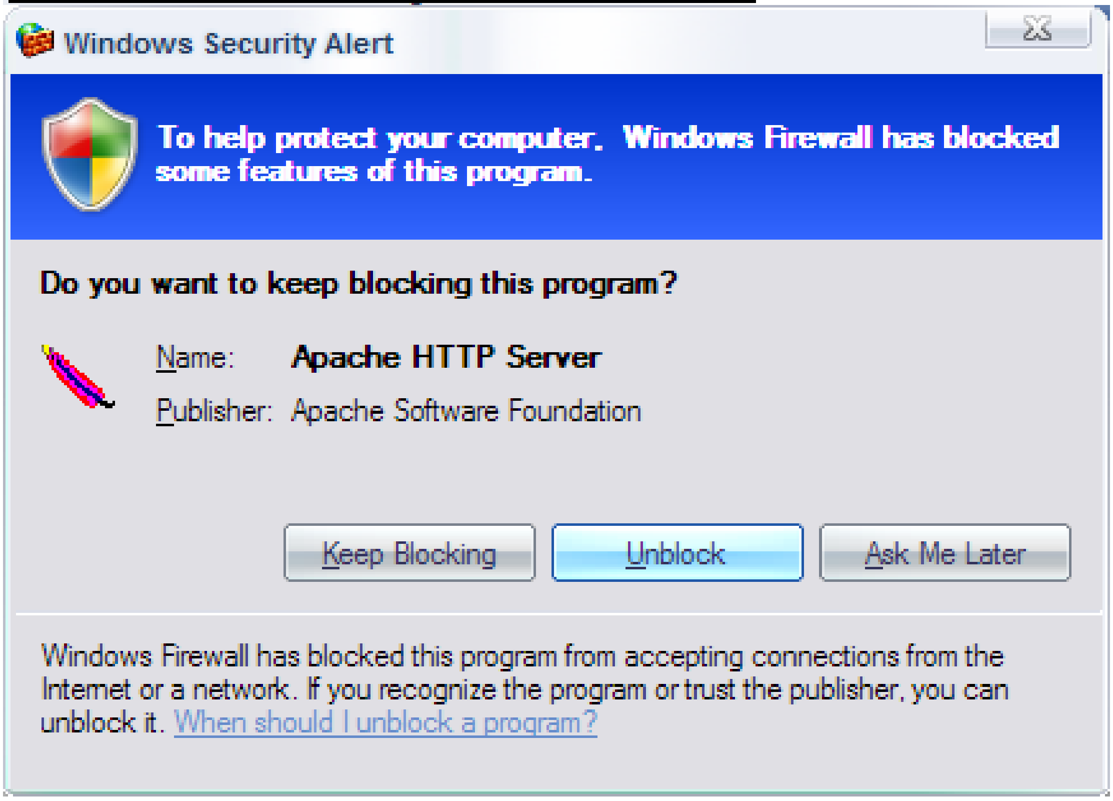

4. Test your installation by running http://localhost/maplab/ (you can replace 'localhost' with your machine's IP address if you know it; Internet users anywhere in the world will be able to access your server using this IP address). You should see the page shown in the screenshot below.

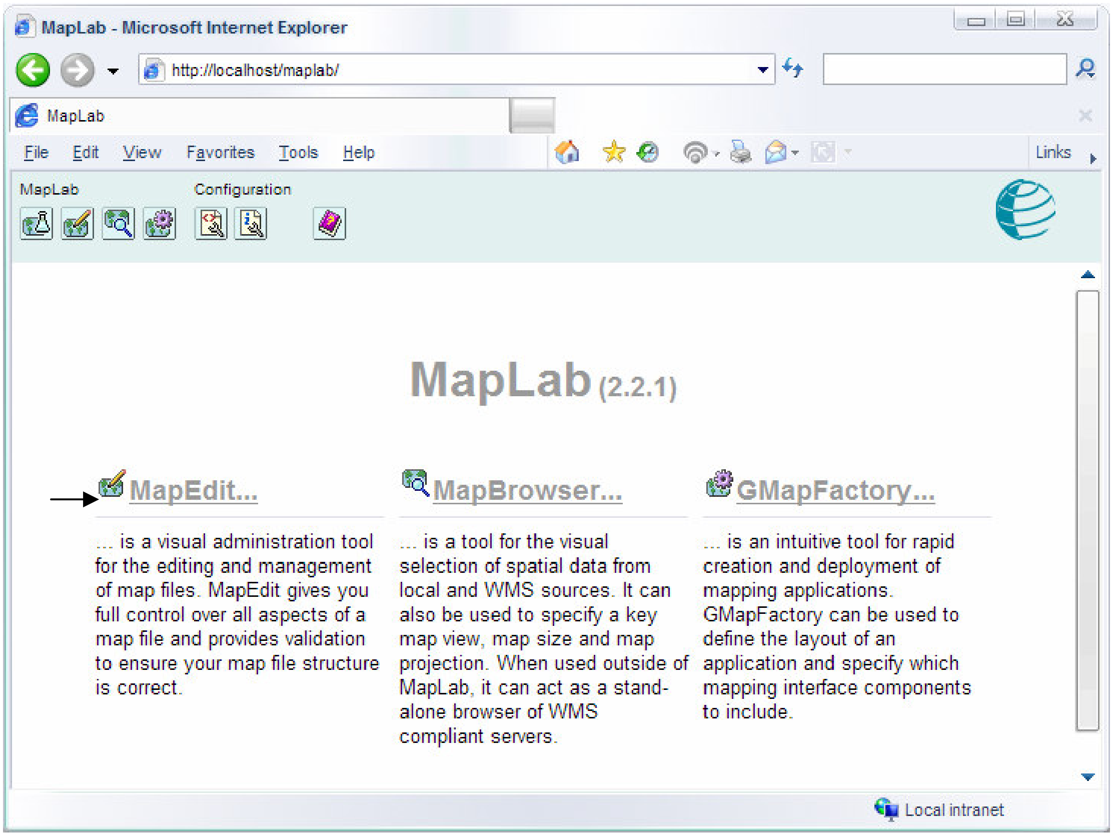

5. Click 'MapEdit' (heading of first column) on the Web page that appears. MapEdit will be displayed. From the 'File Menu' drop-down list choose 'Open Map'. In the 'Open file' box that appears browse to '/maplab-2.2.1/tutorial/', and select and open 'tutorial.map'.

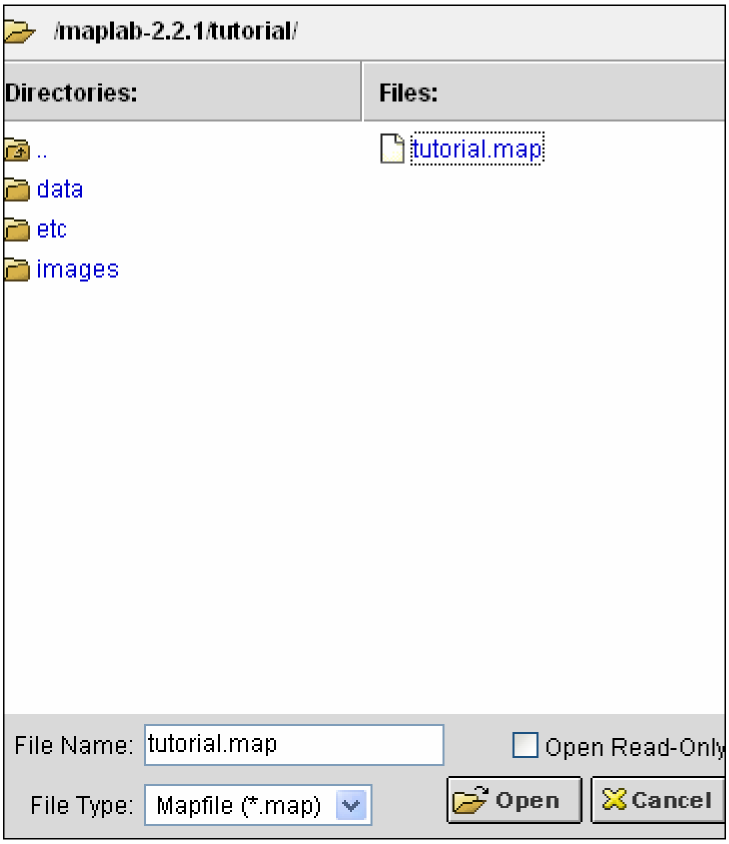

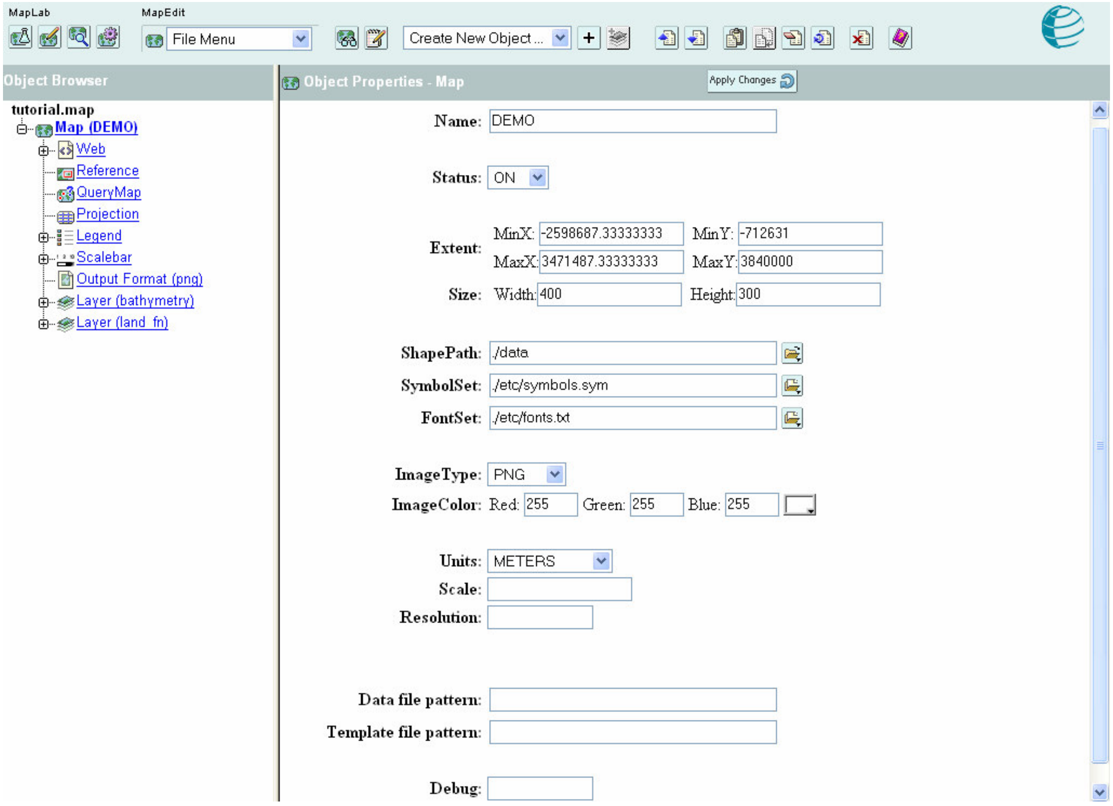

6. The 'tutorial.map' you have just opened is actually a collection of ordinary ESRI Shapefile layers, which you can browse at 'C:\ms4w\apps\maplab-2.2.1\tutorial\data\' on your server's hard drive, and can also open/view in ESRI ArcView or any compatible program.

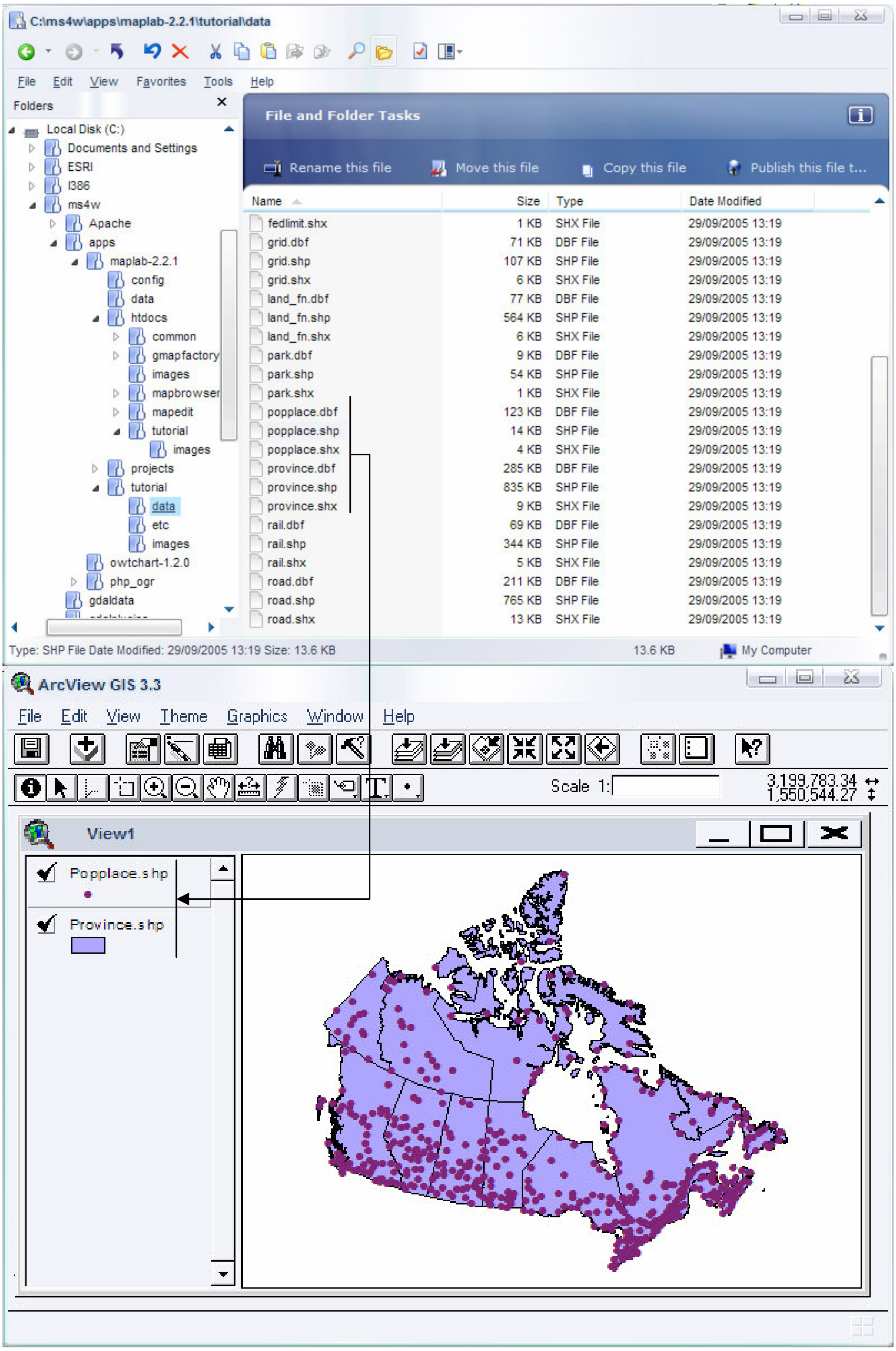

7. The 'tutorial.map' you have just opened is the default 'Canada tutorial' that ships with the MapLab installation ('maplab_ms4w-2.2.1.zip'). There is no map projection defined for it, and as we are planning in this tutorial to access layers from a remote WMS (Web Map Service) server/source and add them to our tutorial map (see below), we will have to add a projection for this WMS step to work.

Adding a Projection Object to the map we have opened in MapEdit: While '**Map (DEMO)**' is selected (should appear in bold font in the 'Object Browser' navigation tree on the left; if not click 'Map (DEMO)' in the navigation tree to select it), go to the drop down menu in the toolbar and change 'Create New Object' to 'Projection Object'. Click on the 'Add New Item (**+**)' button. A 'Projection Object' should appear underneath 'QueryMap' and above 'Legend' in the 'Object Browser' navigation tree on the left. Add the following code in the 'Project Attributes' box: **init=epsg:42304** This information says that we want our map of Canada to be in the Lambert Conformal Conic (LCC) projection for Canada. Next select the 'Save Map' option from the 'File Menu' dropdown.

We are now ready to go to MapBrowser. Click on the 'MapBrowser' button 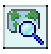 in the toolbar (third button from the left).

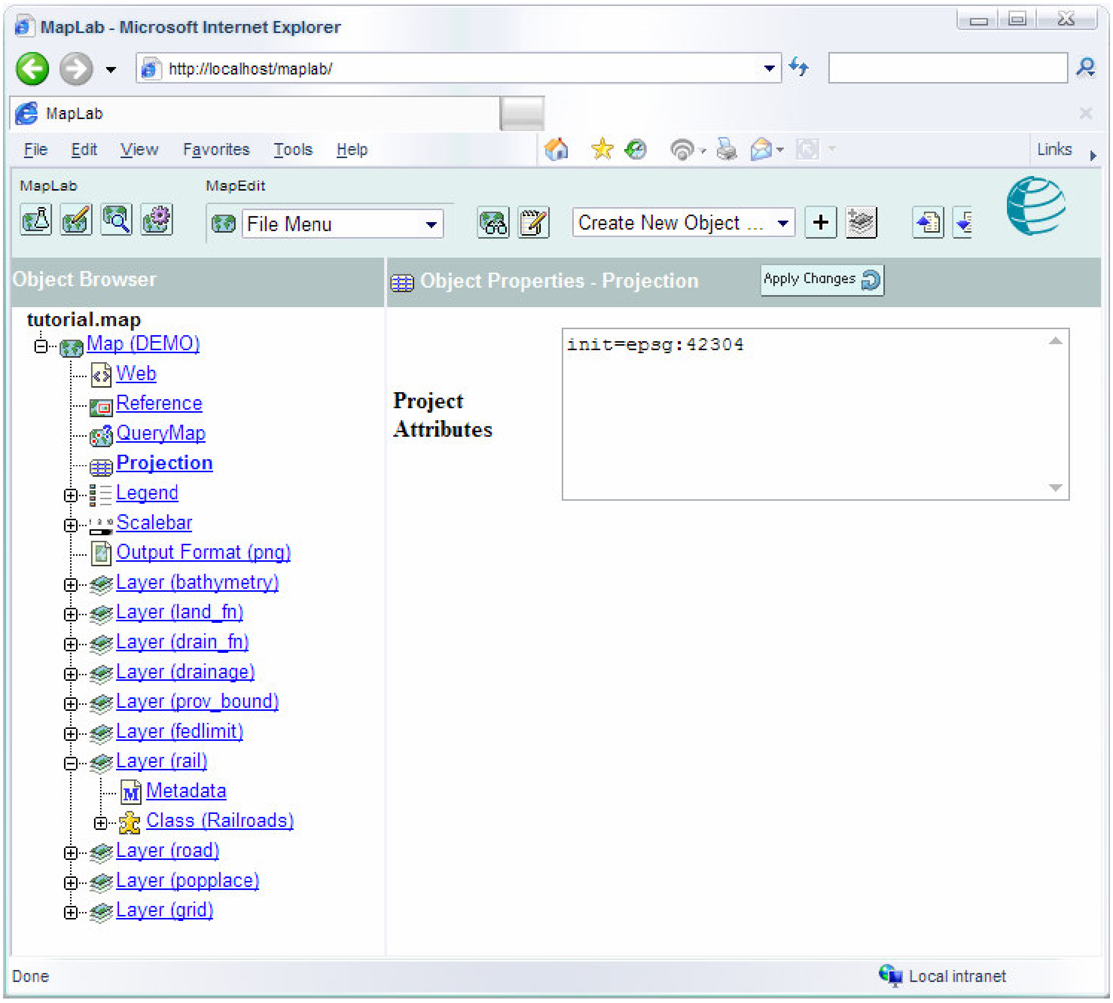

8. MapBrowser will be displayed.

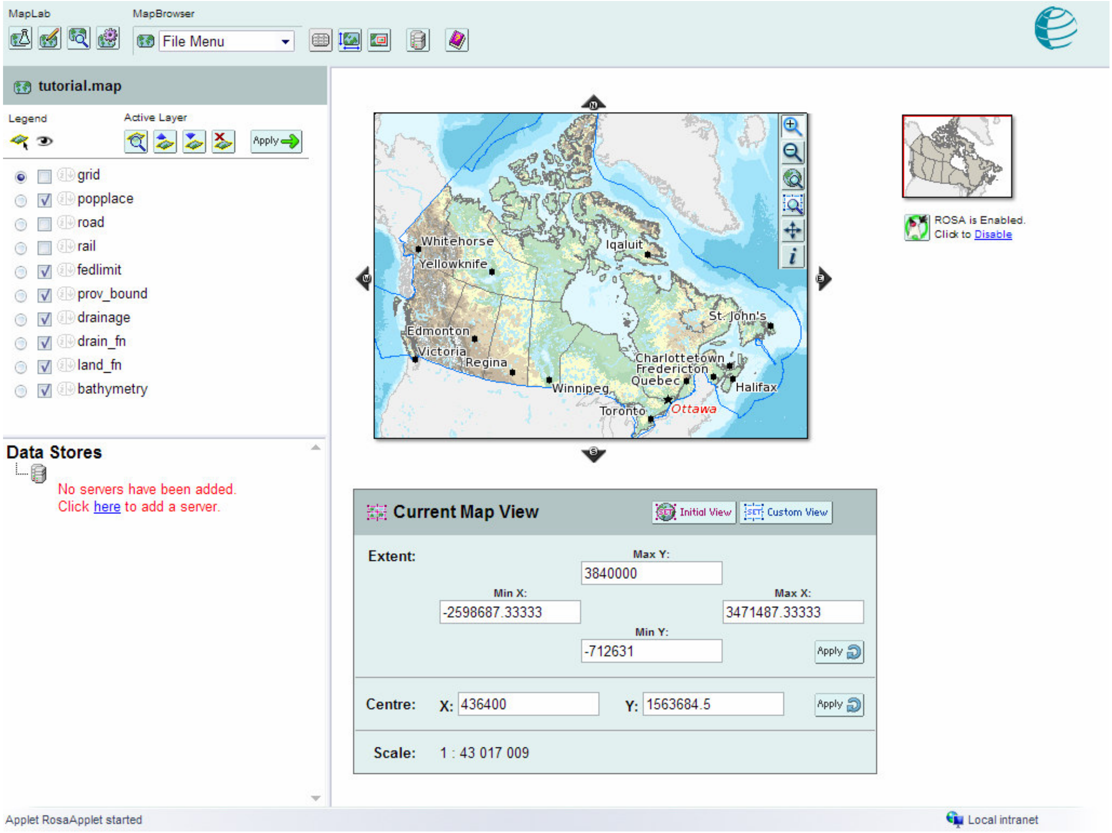

Now in MapBrowser let's zoom into the 'Canadian Province of New Brunswick' (capital is 'Fredericton')/set the 'Current Map View' to it as this is the map area covered by the health layers we are going to import from our chosen remote WMS server (after connecting to it - see below). Either use the map navigation buttons to do so (zoom into 'Fredericton'), or enter the following values in the 'Current Map View' box as show in the screenshot below (don't forget to click 'Apply' after entering the new figures).

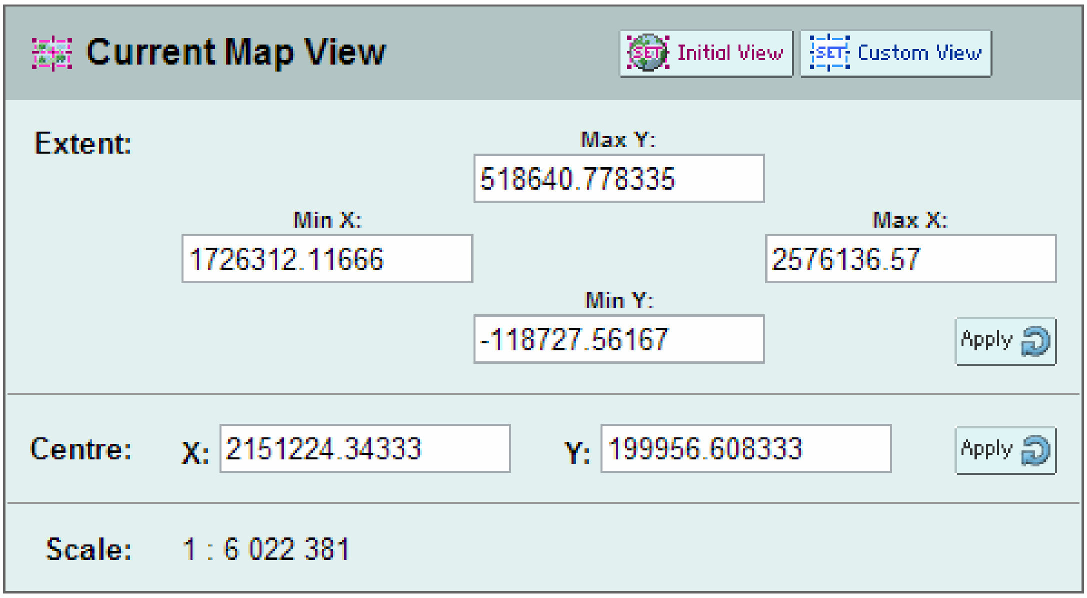

9. Adding our WMS server/source: In MapBrowser, click the 'Manage Servers' button 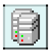 (second button in the toolbar from the right) to add a WMS server or use the link in the lower left pane ('Data Stores') to open the 'Manage Servers' box. In the Manage Servers box, enter **http://156.34.150.115/nbla/servlet/FusionServlet?** in the 'URL' box (under 'Server Properties') and click '+ Add'. After the server is queried and successfully added/connected to, click 'Close' to close the box.

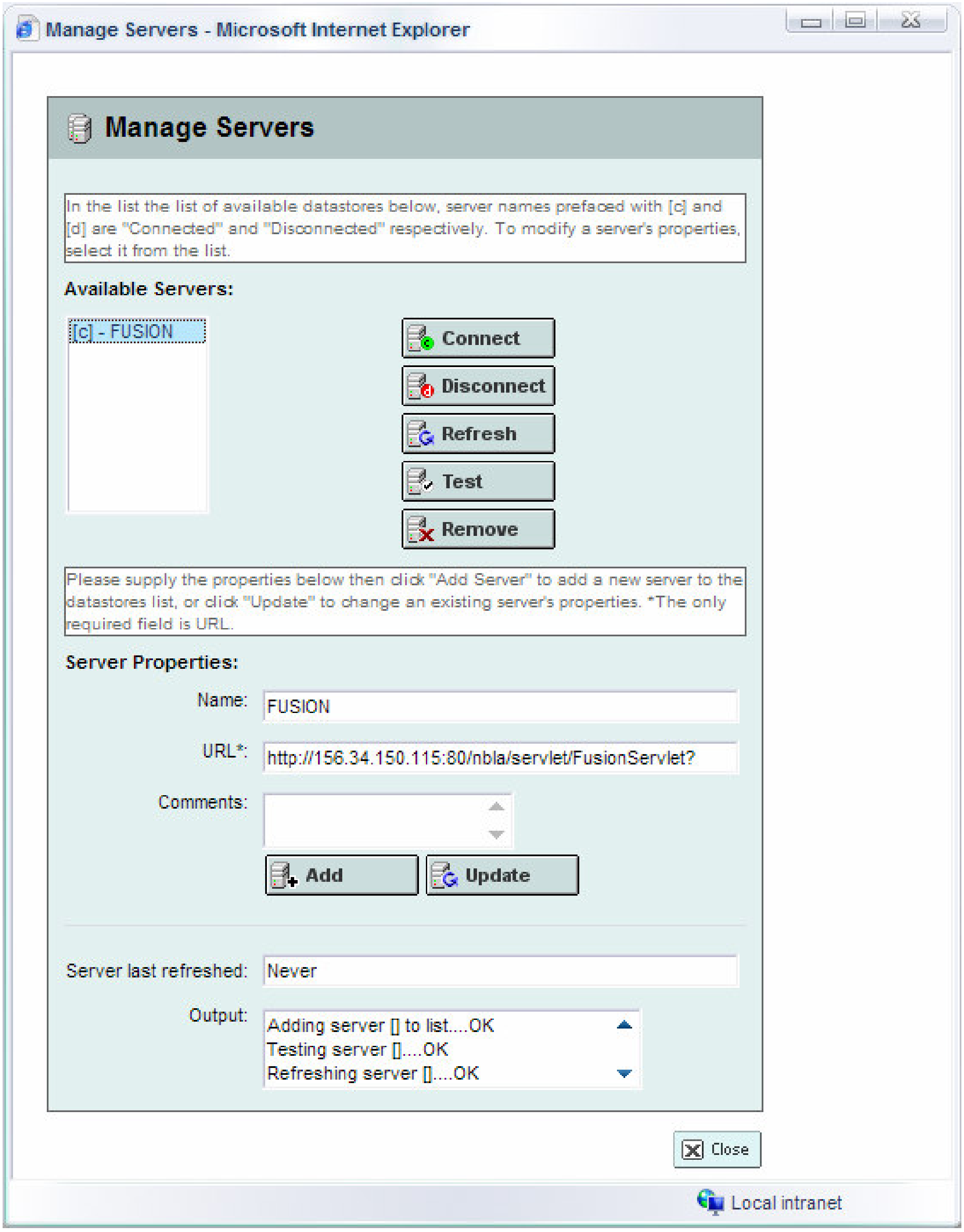

The WMS source we have just connected to is the 'Environmental Health Mapping Portal WMS', which is hosted by the New Brunswick Lung Association; more information about this WMS server can be found at http://www.nb.lung.ca/mapping/ and http://geodecouverte.icdg.ca/gdp/search?action=entrySummary&entryType=service&entryId=5157&entryLang=en&portal=gdp

9. Now back to the main MapBrowser page under 'Data Stores' you should see the new WMS server we have just added and the layers on offer from that server/source. In the WMS server tree, click the branch titled 'healthregion', then click 'Health Regions 2003' layer to select it. The selected layer 'Health Region 2003' should now appear in the upper left pane. If not checked, tick the 'Layer Visibility' box beside it, then click 'Apply' in the upper left pane to add it to/refresh the current map, and you should see the new layer (retrieved from the remote WMS server we have connected to) added to the map on the right.

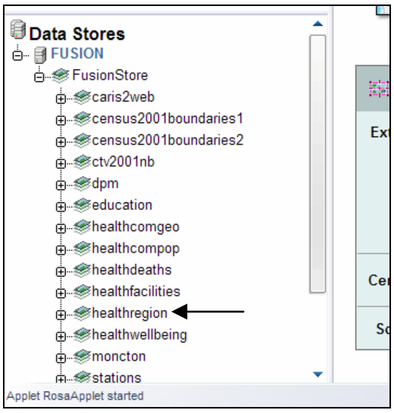

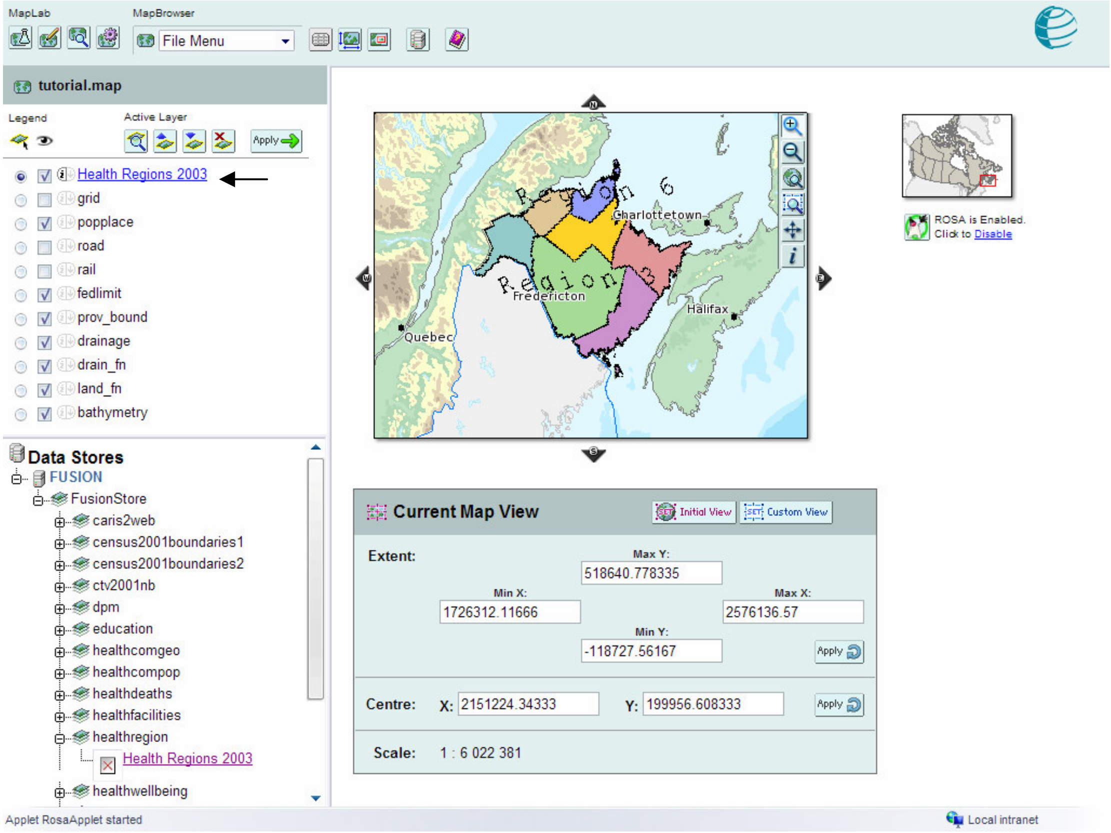

You can click on 'Health Region 2003' layer in the upper pane to display the 'Layer info' box.

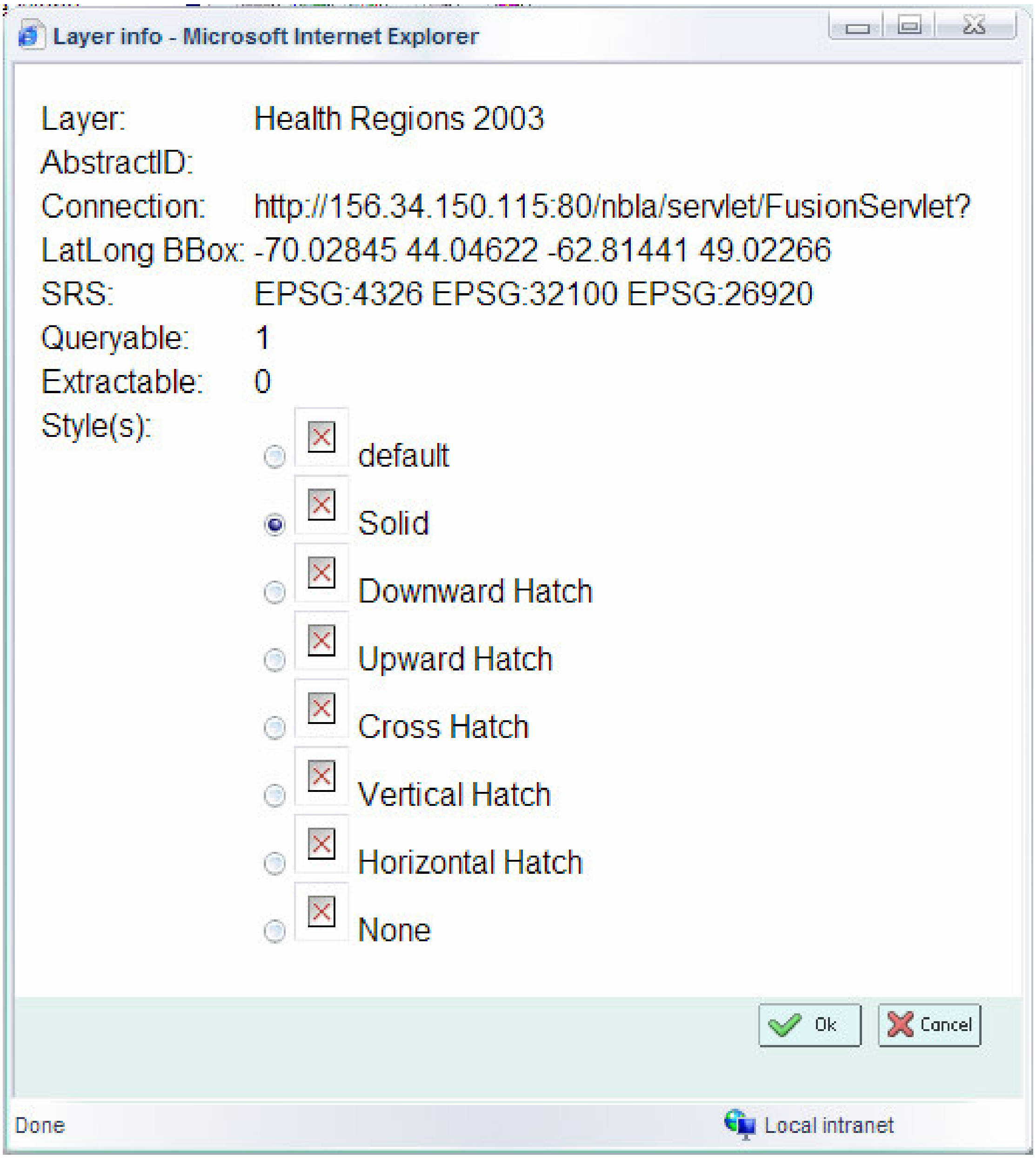

10. Publishing the map: Now click on the 'GMapFactory' button (fourth toolbar button from the left), and in the page that displays type a project name in the 'project' box (e.g., **MyFirstMap**) and select 'Ok'.

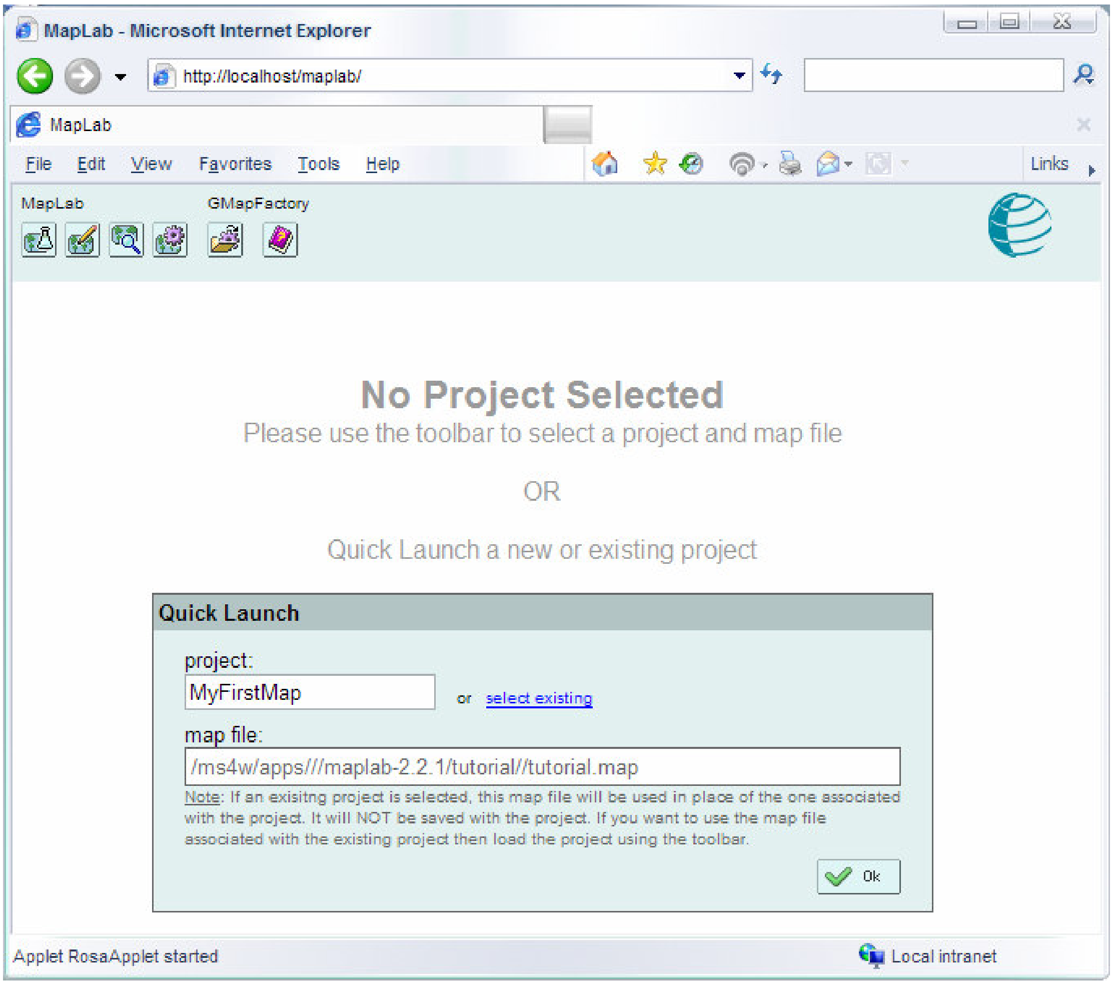

11. The next page will allow you to edit some map settings before publishing it, e.g., change 'Map Title' from 'Untitled' to 'The Canadian Province of New Brunswick' or change the 'Mapping Tools' used from 'ROSA Java-Applet Tools' (more info about ROSA at http://www.maptools.org/rosa/) to 'Standard HTML Tools' (remember to always click 'Apply' 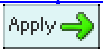

to apply your changes). The page will also give you the URL ('MyFirstMap URL') where this map will be published.

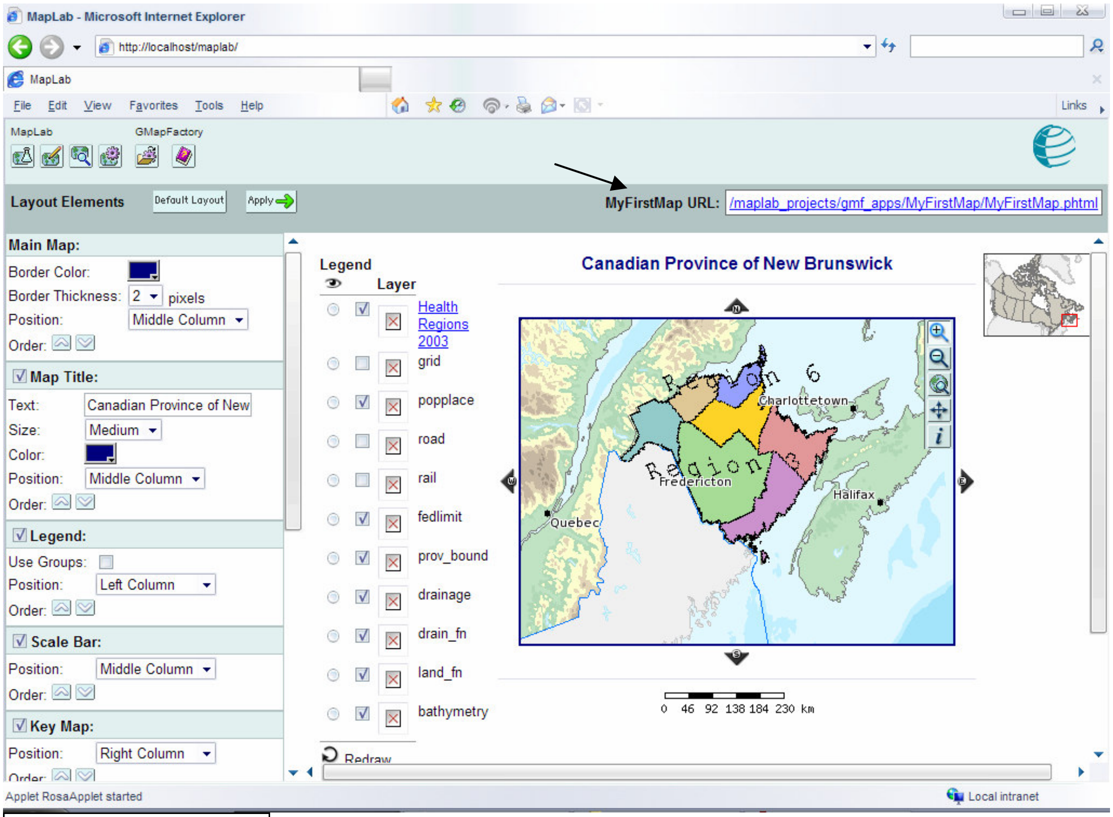

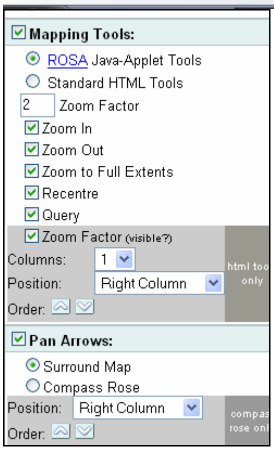

12. Assuming you have named your GMapFactory project 'MyFirstMap' (in step 10 above), the published map should be available for browsing at http://localhost/maplab_projects/gmf_apps/MyFirstMap/MyFirstMap.phtml - To allow Internet users anywhere in the world to connect to your server and see your published map, you need to replace 'localhost' in the previous URL with your Internet-connected machine/server's IP address or domain name, and give them this address.

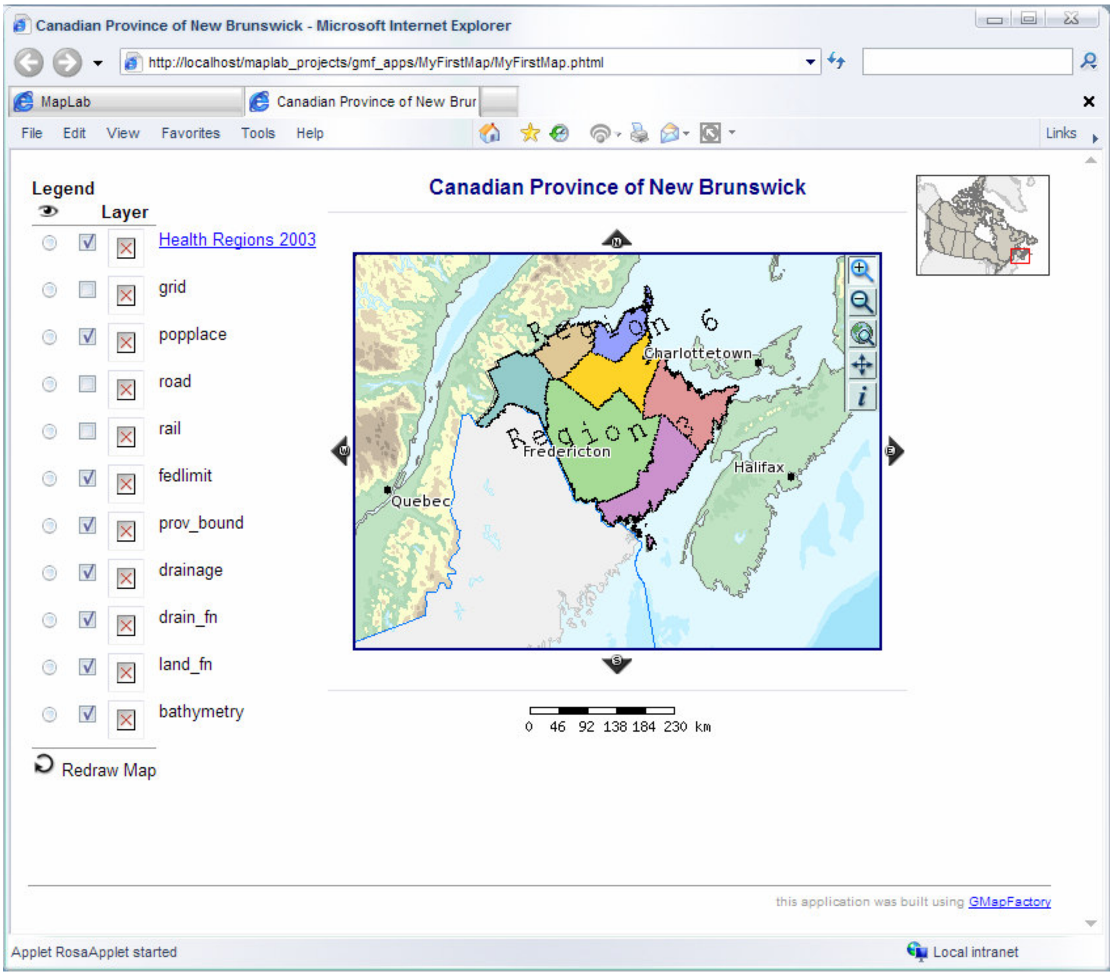

The published interactive map at http://localhost/maplab_projects/gmf_apps/MyFirstMap/MyFirstMap.phtml

Please note that the installation comes with a complete MapLab tutorial that can be accessed at http://localhost/maplab/tutorial/maplab_tutorial.html

The Apache server can be stopped by running 'C:\ms4w\apache-uninstall.bat' – to return your Windows machine to its state prior to this tutorial, simply stop the Apache server then delete the folder 'C:\ms4w' and all its content.

Besides MapLab you can also download and install other applications specially packaged for MS4W, e.g., Chameleon (http://www.dmsolutions.ca/technology/chameleon.html); these can be downloaded at http://www.maptools.org/ms4w/index.phtml?page=downloads.html

Extract any such packages to C:\ or root of the drive where MS4W folder is present, as you did for MapLab ('maplab_ms4w-2.2.1.zip') in step 2 above. The Applications list at http://localhost/ will be automatically updated to list any newly installed packages. Please note that you will need to stop and restart the Apache server in order to use newly installed additional applications.

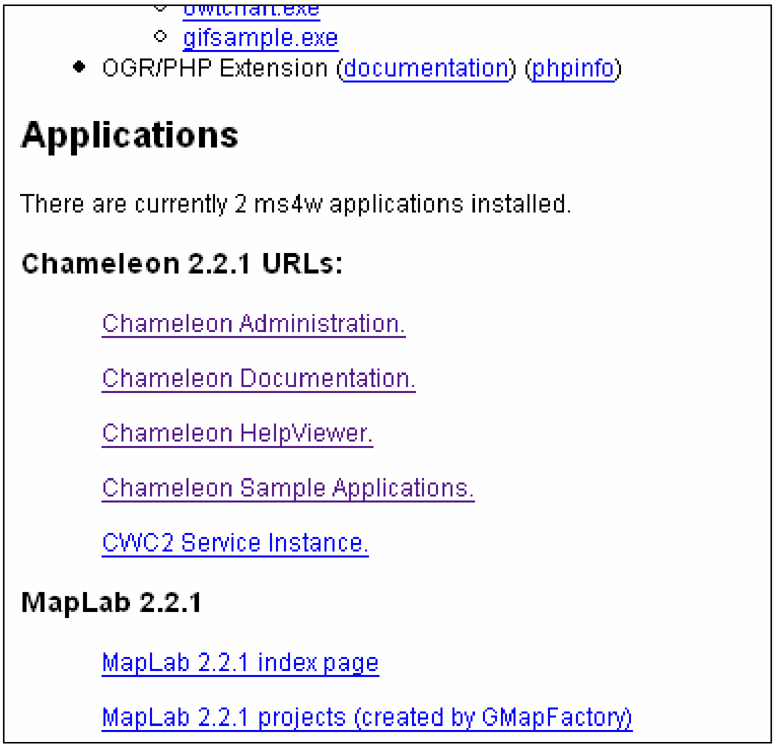

List of Applications at http://localhost/
